# Multiparametric Flow Cytometry Panel for Characterization of Mouse T Cell Differentiation and NK Cell Maturation Following Inflammatory Challenge

**DOI:** 10.3390/mps9030097

**Published:** 2026-06-12

**Authors:** Tim Bozic, Bostjan Markelc, Simona Kranjc Brezar, Ziva Pisljar, Tanja Jesenko, Maja Cemazar

**Affiliations:** 1 Department of Experimental Oncology, Institute of Oncology Ljubljana, Zaloska cesta 2, SI-1000 Ljubljana, Slovenia; tbozic@onko-i.si (T.B.); bmarkelc@onko-i.si (B.M.); skranjc@onko-i.si (S.K.B.); zpisljar@onko-i.si (Z.P.); tjesenko@onko-i.si (T.J.); 2 Biotechnical Faculty, University of Ljubljana, Jamnikarjeva ulica 101, SI-1000 Ljubljana, Slovenia; 3 Faculty of Medicine, University of Ljubljana, Vrazov trg 2, SI-1000 Ljubljana, Slovenia; 4 Faculty of Health Sciences, University of Primorska, Polje 42, SI-6310 Izola, Slovenia

**Keywords:** flow cytometry, immunophenotyping, mouse lymph nodes, T cell differentiation, NK cell maturation

## Abstract

Lymph nodes are central hubs of immune regulation and coordination, serving as primary sites for antigen presentation, lymphocyte activation, and the orchestration of adaptive immune responses. The composition and activation state of lymph node-resident immune cells critically shape both local and systemic immunity. Comprehensive immunophenotyping of these populations is therefore essential for understanding immune organization and functional heterogeneity. Here, we present an optimized protocol for the characterization of mouse lymph node-associated immune populations using 14-color multiparametric flow cytometry. The method combines lymph node isolation based on anatomical landmarks with mechanical dissociation and enzymatic digestion to generate high-quality single-cell suspensions suitable for downstream analysis. Furthermore, the described flow cytometry panel and gating strategy enable reliable identification and quantification of major lymphoid subsets, including helper CD4^+^ and cytotoxic CD8^+^ T cells with their differentiation states, as well as natural killer (NK) cells across distinct maturation stages. Although optimized for assessing lymphocyte maturation after lipopolysaccharide (LPS) challenge, the protocol serves as a reproducible platform for broad immunophenotyping of T and NK cell subsets in mouse lymphoid tissues under experimental conditions.

## 1. Introduction

Lymph nodes are specialized secondary lymphoid organs that play a central role in immune surveillance and regulation [1]. The lymph node comprises a complex network of lymphatic sinuses surrounding a dense parenchyma, which mainly consists of immune cells but also specialized blood vessels and a network of mesenchymal cells [2]. They serve as central hubs where antigens are captured and presented, naïve lymphocytes are activated, and adaptive immune responses are initiated and shaped [3]. The spatial organization and cellular composition of lymph nodes are tightly regulated, and changes in the abundance or activation state of resident immune cells often reflect ongoing physiological or pathological processes [4]. Consequently, precise and reproducible immunophenotyping of lymph node immune populations is fundamental to both basic immunology and translational research.

Among lymphoid cells, T lymphocytes and natural killer (NK) cells are key contributors to immune defense [5]. T cells mediate antigen-specific adaptive immunity, whereas NK cells provide rapid, antigen-independent cytotoxic responses and cytokine production [6]. Both populations display substantial phenotypic and functional heterogeneity, which can be resolved using multiparametric flow cytometry [7]. Mature helper CD4^+^ and cytotoxic CD8^+^ T cells express a functional T cell receptor (TCR) complex composed of CD3 and associated signaling molecules and are identified within the leukocyte compartment by their expression of CD45 [8]. Their differentiation from naïve to effector and memory states is commonly characterized by using the surface markers CD62L and CD44 [9,10]. The adhesion molecules L-selectin CD62L and the glycoprotein CD44 play important roles in T cell trafficking, activation, and differentiation and are critical for the generation and maintenance of immunological memory [11]. Based on their expression patterns, mature T cell populations can be subdivided into naïve (CD62L^+^CD44^−^), central memory (CD62L^+^CD44^+^), effector memory (CD62L^−^CD44^+^), and effector (CD62L^−^CD44^−^) subsets [11]. These phenotypic distinctions are essential for assessing immune activation, antigen experience, and tissue-specific immune organization [12].

NK cells, traditionally classified as innate lymphocytes, also undergo progressive maturation and functional specialization [13]. Murine NK cell development initiates primarily in the bone marrow and continues in peripheral tissues such as the spleen, liver, and lymph nodes, where local microenvironments further shape their phenotype and activity [14,15,16]. NK cell maturation is accompanied by dynamic changes in surface receptor expression, which can be exploited to define discrete developmental and functional stages. In recent years, the concept that NK cells can acquire memory-like features following certain stimuli has expanded our understanding of innate lymphocyte plasticity [6,17]. While such properties are not a defining characteristic of all NK cell responses, their existence underscores the importance of precise phenotypic characterization across NK subsets.

In mice several key markers are associated with NK cell maturation and functional status, including NKp46, CD122, CD244, CD49b, and Ly-49C [18]. These markers are expressed at distinct developmental stages and collectively reflect lineage commitment, maturation progression, and functional competence of NK cells. NK cell development originates from common lymphoid progenitors (CLPs), an early hematopoietic population lacking mature lineage markers (Lin^−^), which give rise to multiple lymphoid lineages, including progenitor B cells, precursor T cells, innate lymphoid cells (ILCs), lymphoid tissue inducer cells, and early NK progenitors [13]. Expression of interleukin-7 receptor-α (IL-7Rα or CD127) within Lin^−^CD244^+^ progenitors marks an early step in lymphoid lineage specification arising from CLPs. Within this population, acquisition of CD122 (the IL-2/IL-15 receptor β chain) defines pre-NK cell precursors (pre-NKPs) progressing toward committed NK progenitors (NKPs) [19]. Together, acquisition of CD244 and CD122 characterizes early NK lineage commitment prior to full maturation [20].

As NK cells mature, they progressively acquire receptors indicative of late-lineage identity, increased functional capacity, and, at later stages, memory-like differentiation. One of the earliest receptors acquired is NKp46 (the mouse homolog of human Natural Cytotoxicity Triggering Receptor 1 (NCR1, also known as CD335)), which serves as a defining NK lineage receptor and plays a key role in target recognition and activation of cytotoxic responses [21,22]. Importantly, NKp46 is a specific NK cell marker in BALB/c mice, where it provides reliable identification of NK lineage cells across developmental stages [23]. Later maturation is also accompanied by the expression of adhesion and integrin molecules. CD49b (DX5), an integrin α chain (VLA-2α), appears during early stages of mature NK cell differentiation and is widely used as a marker of mature conventional NK cells [24]. The acquisition of Ly-49 receptors further defines mature NK cells and their functional competence. Expression of Ly-49C is associated with functionally licensed NK cells capable of appropriate self-recognition and response to target cells [25]. However, not all Ly-49 receptors are equally required for NK cell function. For example, Ly-49G has been reported to be dispensable in certain mouse models, whereas Ly-49C remains strongly associated with mature, licensed NK populations [26].

For both T cells and NK cells, progression toward terminal maturation is associated with increased expression of CD11b (Mac-1) and the terminal differentiation marker killer cell lectin-like receptor G1 (KLRG1) [27,28,29]. In T cells, KLRG1 expression is commonly linked to late differentiation and effector commitment with reduced proliferative capacity and limited ability to generate certain memory subsets [30,31]. However, recent evidence suggests that KLRG1+ T cells may remain highly functional effector cells capable of long-term persistence [32]. In NK cells, maturation is similarly reflected by dynamic changes in surface phenotype of these two markers [13,33,34,35]. In particular, CD11b expression enables discrimination of sequential maturation stages, with CD11b^+^ NK cells representing the most mature subset [13]. Thus, coordinated assessment of CD11b and KLRG1 provides a useful framework for identifying late differentiation states across both adaptive and innate lymphocyte compartments.

These maturation and activation states can be experimentally monitored following lipopolysaccharide (LPS) stimulation, which induces systemic inflammation and activates both innate and adaptive immune responses [36,37,38]. LPS therefore represents a well-characterized experimental stimulus for inducing immune activation and phenotypic remodeling. Although numerous flow cytometry panels have been developed for characterization of either T cell differentiation or NK cell maturation, these approaches typically focus on a single lymphocyte compartment. Consequently, they do not permit simultaneous assessment of adaptive and innate lymphocyte responses within the same sample. To capture these dynamic developmental and functional transitions, we designed a 14-color flow cytometry immunophenotyping panel incorporating described markers that reflect lineage identity, differentiation stage, and activation status. Multiparametric flow cytometry remains one of the most powerful approaches for resolving immune cell heterogeneity at single-cell resolution, enabling simultaneous analysis of multiple markers within complex tissues. However, meaningful interpretation of cytometric data depends critically on standardized sample preparation, validated antibody panels, and well-defined gating strategies. This is particularly relevant for lymph nodes, where cell yields are limited, and specific subsets may occur at low frequencies.

**Figure 1:**
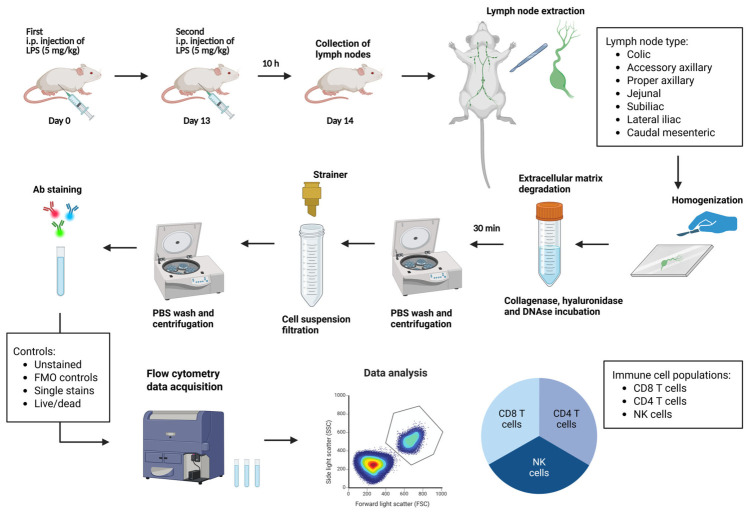
FC-based identification of selected immune cell populations following intraperitoneal (i.p.) lipopolysaccharide (LPS) administration. Schematic overview of the experimental workflow used to identify immune cell populations by FC. Mice received i.p. LPS administration at a dose of 5 mg/kg on day 0 and a second dose of 5 mg/kg on day 13. Lymph nodes were collected on day 14 and mechanically homogenized, followed by enzymatic digestion with collagenase and hyaluronidase. Cell suspensions were subsequently washed and subjected to antibody labeling for surface marker detection. Labeled cells were analyzed by FC, and the resulting data were processed for identification and quantification of selected immune populations. LPS—lipopolysaccharide; i.p.—intraperitoneal; PBS—phosphate-buffered saline; Ab—antibody; FMO—fluorescence minus one.

Here, we present a robust and reproducible protocol for preparing single-cell suspensions from mouse lymph nodes optimized for multicolor flow cytometric analysis (summarized in Figure 1). We describe a flow cytometry panel and gating strategy designed to simultaneously characterize both T cell differentiation states using CD62L and CD44, CD11b and KLRG1 and NK cell maturation stages using CD122, CD11b, NKp46, CD244, CD49b, KLRG1 and Ly-49C. To our knowledge, this specific combination of markers enabling concurrent assessment of CD4^+^ and CD8^+^ T cell differentiation together with sequential NK cell maturation stages within a single assay has not been previously described. The protocol is suitable for the analysis of immune composition under inflammatory and immune-modulating conditions and in a variety of experimental settings, providing a versatile framework for immunological studies focused on coordinated T and NK cell responses.

## 2. Experimental Design

### 2.1. Animals

Immunocompetent 6–8-week-old BALB/cAnNCrl female mice weighing between 18 and 20 g (Charles River International, Boston, MA, USA). All the animal experiments were conducted in accordance with the EU Directives for animal experiments and the ARRIVE Guidelines and were approved by the Ministry of Agriculture, Forestry, and Food of the Republic of Slovenia (permission no. U34401-3/2022/17).

### 2.2. Materials

1. Lipopolysaccharides from Pseudomonas aeruginosa 10 (LPS) (Cat. N. L9143-10MG; Sigma-Aldrich, Darmstadt, Germany).
2. Collagenase IV (1 g) (Cat. N. LS004188; Worthington Biochemical, Lakewood, NJ, USA).
3. Hyaluronidase (100 mg) (Cat. N. H3506; Sigma-Aldrich, Darmstadt, Germany).
4. DNase (20 U/µL)—Innuprep DNase I digestion kit (Cat. N. 845-KS-5200250; Analytik Jena, Jena, Germany).
5. BD Horizon™ Brilliant Stain Buffer (Cat. N. 566349; BD Biosciences, Franklin Lakes, NJ, USA).
6. CellBlox™ Plus Blocking Buffer (Cat. N. C001T03F01; Thermo Fisher Scientific, Waltham, MA, USA).
7. TruStain FcX™ Plus (anti-mouse CD16/32) Antibody (Cat. N. 156604; Biolegend, San Diego, CA, USA).
8. (Optional) Fixation Buffer (IC Fixation Buffer, Cat. N. 00-8222-49, eBioscience, San Diego, CA, USA).
9. (Optional) 10× Red Blood Cell (RBC) Lysis Buffer (Cat. N. 420301, BioLegend, CA, USA).
10. 10× Phosphate-buffered saline (PBS) (Cat. N. 70013032, Thermo Fisher Scientific, MA, USA).
11. 1× Hanks’ Balanced Salt Solution (HBSS) buffer (Ca^2+^, Mg^2+^) (Cat. N. 14025050, Gibco™, Thermo Fisher Scientific, MA, USA).
12. Fluorescently labeled antibodies and viability dye for flow cytometry (FC) (see Table 1; store protected from light at 4 °C).
13. Compensation beads for single-stain controls—UltraComp eBeads™ Plus Compensation Beads (Cat. N. 01-3333-42; Thermo Fisher Scientific, MA, USA).
14. Distilled water (dH_2_O) (B. Braun Medical, Melsungen, Germany).
15. Physiological saline (B. Braun Medical, Melsungen, Germany).
16. Ethanol (70%).

### 2.3. Panel Design

The presented flow cytometry panel was adapted and expanded from a panel previously used in the study by Pisljar et al. [39], with additional markers incorporated to enable simultaneous characterization of T cell differentiation and NK cell maturation within a single assay. Marker selection was based on their established utility for defining major CD4^+^ and CD8^+^ T cell differentiation states and sequential NK cell maturation stages.

Fluorochrome assignment was guided by antigen density, expected expression levels, fluorochrome brightness, spectral overlap, detector availability, and the need to minimize spillover into channels used for dim or biologically critical markers. Brighter fluorochromes were preferentially assigned to markers with lower or variable expression, whereas highly expressed lineage markers were assigned to fluorochromes with lower brightness when appropriate. The final panel configuration was optimized using unstained controls, single-stained compensation controls, and biologically defined gating populations to ensure reliable resolution of all the targeted cell subsets.

### 2.4. Reagent Preparation

- LPS:

Prepare LPS in physiological saline (0.9% NaCl) at a concentration of 1 mg/mL.

- Homogenization Buffer:

First, prepare separate working solutions of collagenase IV (80 mg/mL) and hyaluronidase (32 mg/mL) in HBSS containing Ca^2+^ and Mg^2+^. Next, prepare the digestion buffer mixture by adding the appropriate volumes of the collagenase IV and hyaluronidase working solutions to 5 mL of HBSS (containing Ca^2+^ and Mg^2+^) to obtain final concentrations of 3.2 mg/mL and 0.32 mg/mL, respectively. Add DNase I to a final concentration of 2 U/mL. Keep the buffer at 4 °C until use.

- (Optional) Red Blood Cell (RBC) Lysis Buffer (see Note 1):

Dilute 10× RBC lysis buffer with distilled water (dH_2_O) to obtain a 1× working solution. Prepare fresh before use.

- TruStain FcX™ Plus Mix:

For each sample prepare TruStain FcX™ Plus at a concentration of 0.25 µg per 1 × 10^6^ cells in a total volume of 100 µL of 1× PBS.

- Antibody Master Mix:

Prepare the Antibody Master Mix in 1× PBS to a final staining volume of 100 µL per sample. The mix should contain 5% CellBlox™ Plus, 50% Brilliant Stain Buffer, and all the antibodies according to the dilutions listed in Table 1. Account for the additional 10 µL of Live/dead Staining Mix per sample when calculating final volumes.

- FMO Buffer:

Prepare the FMO Buffer in 1× PBS to a final staining volume of 160 µL per FMO control. The mix should contain 5% CellBlox™ Plus and 50% Brilliant Stain Buffer. Account for the additional antibody volume when calculating final volumes.

- Live/dead Staining Mix:

Prepare the Live/dead Staining Mix immediately before use. For 10 samples, combine 100 µL of 1× PBS with 1 µL of Fixable Viability Dye eFluor^®^ 780. Add 10 µL per sample to Antibody Master Mix just before staining the cells, including the live/dead single-stain control, to reach a total staining volume of 100 µL per sample.

- Fixation Buffer:

Prepare Fixation Buffer by diluting IC Fixation Buffer 1:1 with distilled water immediately before use (see Note 2).

### 2.5. Equipment

- Surgical scissors (Cat. N. 14064-11, Stevens Scissors, Fine surgical tools, Heidelberg, Germany).
- Forceps (Cat. N. 140-228, Ewald-Hudson Tissue Forceps, ELCON Medical Instruments, Tuttlingen, Germany).
- Pipettes and tips.
- Glass cutting board.
- Petri dish.
- A 1 mL syringe plunger.
- Disposable stainless-steel scalpel (Cat. N. PDSS20, Paramount Surgimed Ltd., New Delhi, India).
- 50 µm cell strainer (Cat. N. 04-0042-2317 or 04-004-2327, Sysmex, Hyogo, Japan).
- Cell counting chamber (Cat. N. 07-200-988, Corning™ Counting Chamber, Thermo Fisher Scientific, MA, USA).
- Automated Cell Counter (Axion Biosystems, Atlanta, GA, USA).
- Thermoshaker (Kühner ISF-1-W Incubator Shaker, Kühner, Basel, Switzerland).
- Refrigerated centrifuge with swinging buckets (Centrifuge 5810 R, Eppendorf, Hamburg, Germany).
- BD FACSymphony A3 flow cytometer (BD Biosciences, Franklin Lakes, NJ, USA). The panel may be adapted to other multiparametric flow cytometers with sufficient detector capacity, provided that appropriate fluorochrome compatibility, compensation, and panel optimization are performed. Configuration of the flow cytometer is shown in Table 2 (see Note 3).

### 2.6. Software

- BD FACSDiva™ Software (Becton Dickinson, NJ, USA, v9.1 or later; accessed on 25 February 2026) or any equivalent,
- FlowJo analysis software (BD Biosciences, NJ, USA, v10 or later; accessed on 25 February 2026) or any equivalent.

## 3. Procedure

### 3.1. LPS Administration

Before intraperitoneal LPS injection, the LPS solution should be prewarmed to 37 °C to prevent hypothermic stress upon administration. Following treatment, animals should be monitored at least four times daily for three consecutive days, as this period corresponds to the peak systemic response to LPS. During this acute phase, body weight should be measured every 2–3 h to allow early detection of rapid weight loss and clinical deterioration. Clinical monitoring should include assessment of general appearance, posture, mobility, respiratory pattern, hydration status, and body weight in accordance with the PREPARE [40] guidelines. Any mice that lost more than 20% of their initial body weight without gaining weight within the next 48 h should be humanely euthanized.

To minimize treatment-related mortality (approximately 15%) and support recovery, appropriate supportive measures must be implemented, particularly during the first three days post-injection. Cages should be provided with a controlled external heat source (e.g., an infrared lamp or heating pad placed beneath part of the cage) to allow animals to thermoregulate voluntarily. Care should be taken to avoid overheating and to ensure that animals can move away from the heat source if needed. In addition, 500 µL of 3% glucose solution, prewarmed to 37 °C, should be administered intraperitoneally once daily to provide metabolic support during the acute inflammatory phase. Continued monitoring throughout the experiment is essential, and additional glucose supplementation may be administered at later time points if signs of weakness, weight loss, or reduced intake persist. Supplemental hydration and softened food placed on the cage floor may further improve recovery. All supportive interventions should be performed in accordance with approved animal care protocols and ethical regulations.

### 3.2. Lymph Node Dissociation

Immune cells are highly fragile; therefore, all steps should be performed as quickly and gently as possible. To preserve cell viability, keep cells and reagents on ice or at 4 °C whenever feasible throughout the procedure. All the centrifugation steps must be performed at 4 °C. Minimize handling time and mechanical stress to reduce potential cell activation and cell death. All the buffers described in the “Reagent Preparation” Section 2.4 should be freshly prepared prior to each FC staining.

**Figure 2:**
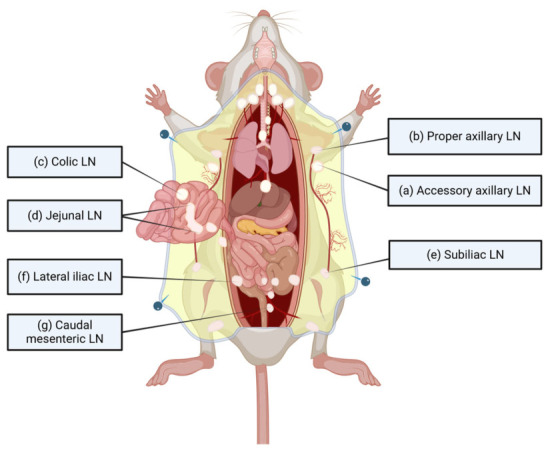
Anatomy of the mouse and schematic representation of lymph node locations relevant to the FC protocol. Schematic overview of mouse anatomy illustrating the anatomical landmarks used for lymph node dissection. The nomenclature and location of lymph nodes (LNs) are based on the description of Van den Broeck et al. [41]. Lymph nodes in normal, untreated mice (e.g., BALB/cAnNCrl) are typically small (approximately 1–2 mm in diameter) and difficult to detect with the naked eye; however, following certain treatments, lymph nodes may enlarge and become easier to identify. The locations of major peripheral and visceral lymph nodes are indicated, including: (**a**) accessory axillary lymph nodes, located cranial to the proper axillary nodes near the shoulder region; (**b**) proper axillary lymph nodes, positioned in the axillary fat pad adjacent to the forelimb; (**c**) colic lymph nodes, associated with the colon along the mesenteric vessels; (**d**) jejunal lymph nodes, distributed along the mesentery of the jejunum; (**e**) subiliac (inguinal) lymph nodes, situated in the inguinal region near the hind limb; (**f**) lateral iliac lymph nodes, located adjacent to the iliac vessels within the pelvic cavity; and (**g**) caudal mesenteric lymph nodes, positioned at the base of the mesentery near the distal colon.

- Euthanize the mouse by performing cervical dislocation.
- Place the mouse in a supine position on a dissection surface.
- Spray the fur generously with 70% ethanol to reduce contamination.
- Use sterile recommended scissors and forceps for dissection.
- Make a midline skin incision. Reflect the skin laterally and fixate using needles to expose underlying tissues.
- Dissect lymph nodes by following the anatomical landmarks (see Figure 2 and Table 3):

(a) Accessory Axillary Lymph Nodes
  - Located superficial and cranial to the axilla, near the base of the forelimb.
  - Gently retract the forelimb laterally.
  - Identify small, pale, oval lymph nodes embedded in subcutaneous fat.
  - Carefully separate the lymph node from surrounding adipose tissue using forceps.
(b) Proper Axillary Lymph Nodes
  - Located deeper within the axilla, adjacent to the brachial vessels.
  - Retract the forelimb fully to expose the axillary cavity.
  - Remove overlying fat to visualize the node.
  - Avoid damaging nearby blood vessels.
(c) Colic Lymph Nodes
  - Open the abdominal cavity by cutting through the peritoneum along the midline.
  - Gently exteriorize the intestines.
  - Locate lymph nodes along the colon and mesocolon, near colic blood vessels.
  - Identify small, firm, pale nodules embedded in mesenteric tissue.
(d) Jejunal Lymph Nodes
  - Found within the mesentery of the jejunum, close to the superior mesenteric vessels.
  - Spread the small intestine gently to expose the mesenteric fan.
  - Identify multiple lymph nodes aligned along the mesenteric vasculature and excise the largest single node, as indicated in Figure 2.
(e) Subiliac (Inguinal) Lymph Nodes
  - Located subcutaneously near the hind limb, anterior to the hip joint.
  - Reflect the skin of the lower abdomen and upper thigh.
  - Identify the lymph node within the inguinal fat pad.
(f) Lateral Iliac Lymph Nodes
  - Located deep in the pelvic region, lateral to the iliac vessels.
  - Gently move the intestines cranially to expose the pelvic cavity.
  - Identify lymph nodes adjacent to the external iliac vessels.
  - Use fine forceps to separate the node from surrounding tissue.
  - Avoid excessive pressure due to proximity to major vessels.
(g) Caudal Mesenteric Lymph Nodes
  - Found near the distal colon and rectum, close to the caudal mesenteric artery.
  - Gently retract the colon to visualize the mesenteric attachment.
  - Identify the lymph node embedded near vascular branching.
  - Excise each located lymph node (11 lymph nodes in total) with minimal traction and transfer immediately to a Petri dish containing physiological saline.
  - Proceed promptly to homogenization and cell isolation to maintain immune cell viability.

### 3.3. Lymph Node Homogenate Processing to Obtain Single Cell Suspension

- Place excised lymph nodes on a clean glass cutting board (see Note 4).
- Add 200 µL of the Homogenization Buffer to the lymph nodes and homogenize them mechanically using two scalpels (see Note 5).
- Transfer the homogenized lymph nodes to a 50 mL centrifuge tube containing 5 mL of Homogenization Buffer. Place the suspension on ice until all the homogenate has been transferred.
- Incubate the suspension for 30 min with shaking (150 rpm) at 37 °C (see Note 6).
- Transfer the suspension through a 50 µm sterile cell strainer placed on top of a 15 mL centrifuge tube.
- Using a 1 mL pipette and a syringe plunger, wash the cell strainer with 5 mL of 1× PBS (see Note 7).
- Centrifuge the filtrate for 5 min at 400× *g* and 4 °C. Discard the supernatant.
- Wash the pellet with 2 mL of 1× PBS and repeat the centrifugation step. Discard the supernatant.
- (Optional) Resuspend the pellet in 5 mL of 1× RBC Lysis Buffer (see Note 1).
  - Incubate on ice for 5 min with occasional shaking.
  - Stop the reaction by diluting 1× RBC Lysis Buffer with 20 mL of 1× PBS.
  - Centrifuge 5 min, 400× g, 4 °C. Discard the supernatant.
- Resuspend cells in 1 mL of 1× PBS and count the cells using an automated cell counter (see Note 8).

### 3.4. Antibody Sample Staining for FC-Based Immunophenotyping

- Prepare labeled polystyrene round-bottom tubes for flow cytometry (FACS tubes), each containing 2 mL of 1× PBS according to Table 4.
- For staining take 3 × 10^6^ cells per sample by transferring the appropriate volume into prepared FACS tubes (see Note 9). The same number of cells should be added to unstained, live/dead single-stain and each fluorescence minus one control (FMO) tube (see Note 10).
- Centrifuge 5 min at 400× *g* and 4 °C. Discard the supernatant.
- Add 100 µL TruStain FcX Plus mix to each FACS tube (see Note 11). To tubes containing unstained cells and live/dead single-stain control, add 100 µL 1× PBS instead.
- Incubate on ice for 5 min.
- Centrifuge 5 min at 400× *g* and 4 °C. Discard the supernatant.
- Separately prepare the Antibody Master Mix (for antibody dilutions, refer to Table 1), FMO Buffer and Live/dead Staining Mix.
- Prepare unstained cells by resuspending them in 100 µL of 1× PBS.
- Prepare FMO controls by mixing antibodies according to the dilutions presented in Table 1 in FMO buffer.
- Prepare single-stain controls.
  - Add 1 drop of compensation beads to each FACS tube.
  - Add the appropriate volume of each membrane marker antibody to the corresponding FACS tube according to the dilutions listed in Table 1, adjusting with 1× PBS to a final volume of 100 µL.
  - In parallel, prepare a positive Live/dead single-stain control by incubating 3 × 10^6^ cells in 2 mL of 1× PBS for 1 min at 65 °C, followed by 1 min on ice. Centrifuge for 5 min at 400× *g* and 4 °C, then discard the supernatant. Resuspend the cells in a total volume of 100 µL consisting of 90 µL of 1× PBS and 10 µL of Live/dead Staining Mix.
- Prepare samples
  - Add the Live/dead Staining Mix to prepared Antibody Master Mix just before adding to the cells.
  - To each FACS tube containing a sample add 100 µL of Antibody Master Mix.
- Incubate FACS tubes with samples, unstained cells, FMO and single-stain controls for 30 min on ice, protected from light.
- Wash cells twice with 1 mL of 1× PBS. For the first wash, add 1× PBS directly to the stained cells.
- After the second wash resuspend stained cells in 400 µL Fixation Buffer.
- Proceed to acquisition or store samples overnight at 4 °C.

### 3.5. FC Gating Strategy and Data Analysis

Before proceeding, confirm that all the required sample types are prepared to ensure a successful FC experiment. These should include: immunophenotyping samples, unstained control, FMO controls, positive live/dead single-stain control (dead cells), single-stain controls for compensation matrix (set up using compensation beads) (see Table 4).

**Figure 3:**
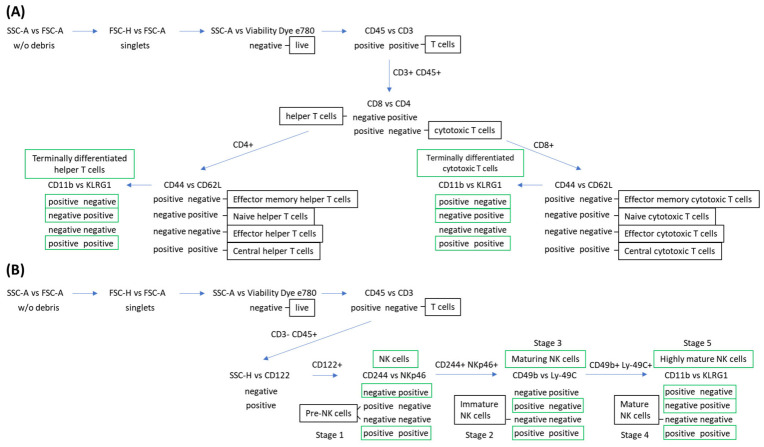
Schematic representation of the gating strategy for FC data acquisition. Panel (**A**) shows the gating strategy of T cells, while panel (**B**) shows the gating strategy of NK cells. FSC-A/H (forward scatter area/height) and SSC-A/H (side scatter area/height) were used for initial cell selection and doublet exclusion. Arrows indicate the sequential gating steps and the markers used for population delineation. Marker combinations are displayed below each step as binary states as positive/negative. Binary states highlighted in green rectangles correspond to the immune populations in green rectangles indicated above the markers. Binary states without rectangles correspond to populations indicated in adjacent black rectangles. The populations shown above the arrows represent the gated subsets defined by the preceding marker combinations.

**Figure 4:**
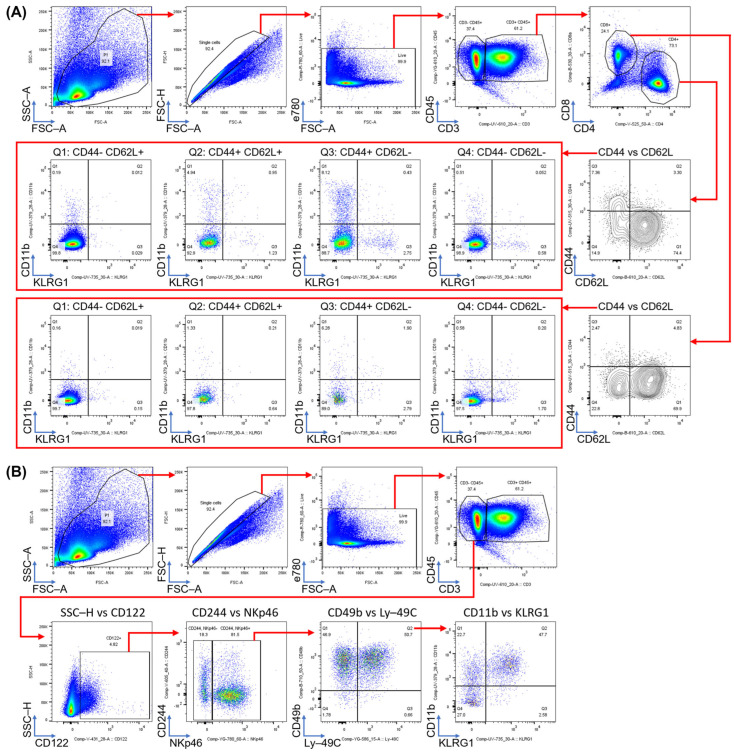
Gating strategy used for identification of immune cell populations. The presented gating strategy was adapted from Abel et al. [13] and illustrates sequential identification of T and NK cell populations. Panel (**A**) shows the sequential gating strategy for T cells, including selection of P1 events, singlets, and live cells, followed by separation into CD3^−^CD45^+^ and CD3^+^CD45^+^ fractions. Within the CD3^+^CD45^+^ compartment, CD4^+^ (helper) and CD8^+^ (cytotoxic) T cells are identified. Each population is further subdivided based on CD44 and CD62L expression into four differentiation states: naïve (CD62L^+^CD44^−^), central memory (CD62L^+^CD44^+^), effector memory (CD62L^−^CD44^+^), and effector (CD62L^−^CD44^−^) T cells. These subsets are defined using quadrant gating (Q1–Q4) on CD44 versus CD62L plots. Each quadrant is subsequently analyzed for terminal differentiation by assessing CD11b and KLRG1 expression, allowing identification of more differentiated effector populations within each subset. Panel (**B**) shows the same initial gating strategy as in Panel (**A**) (P1 events, singlets, live cells, and separation into CD3^−^CD45^+^ and CD3^+^CD45^+^ fractions), followed by focused analysis of the CD3^−^CD45^+^ compartment. NK cells are identified within this compartment based on lymphocyte scatter properties and expression of the precursor-associated marker CD122, followed by CD244 and NKp46 expression to define NK lineage cells. This population is further stratified by acquisition of CD49b and Ly49C, allowing discrimination of maturing and mature NK cell subsets. Finally, expression of CD11b and KLRG1 is used to identify terminally differentiated NK cell subpopulations. In both panels, red arrows indicate the sequential gating progression from parent to downstream populations.

**Figure 5:**
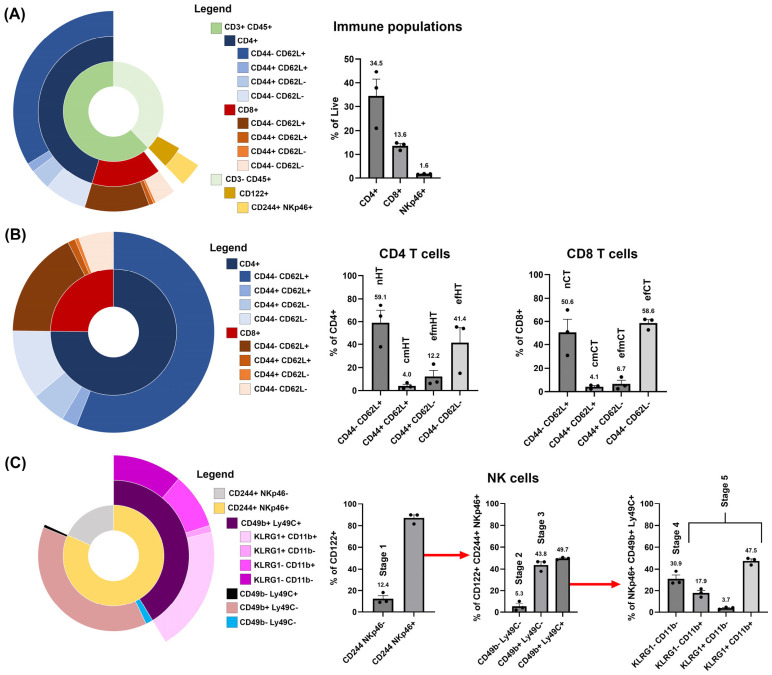
Example representation of immune cell populations identified using the proposed flow cytometry panel. Pie charts illustrate a representative output of the proposed immunophenotyping workflow using lymph nodes collected 14 days after the initial i.p. LPS administration. In panel (**A**) the inner circle represents the relative proportions of CD3^−^CD45^+^ and CD3^+^CD45^+^ cells, expressed as frequencies of live cells and normalized to 100% of total CD45^+^ leukocytes. The middle ring shows the distribution of major lymphocyte populations within these compartments: CD4^+^ and CD8^+^ T cells within the CD3^+^CD45^+^ compartment, and CD122^+^ cells within the CD3^−^CD45^+^ compartment. The outer ring further subdivides CD4^+^ and CD8^+^ T cells into naïve, central memory, effector memory, and effector subsets. NK cells (CD244^+^NKp46^+^) are also included and shown as percentages of their respective parent populations. Bar graphs display CD4^+^, CD8^+^, and NKp46^+^ cells as percentages of live cells. In panel (**B**) the T cell compartment is shown separately. The inner circle displays the relative proportions of CD4^+^ and CD8^+^ T cells, expressed as frequencies of live cells and normalized to 100%. T cell subsets are presented as percentages of their respective parent populations. Bar graphs show the proportions of memory and effector CD4^+^ (helper) and CD8^+^ (cytotoxic) T cell populations. In panel (**C**) the NK cell compartment is shown separately. The inner circle displays the relative proportions of CD244^+^NKp46^+^ and CD244^+^NKp46^−^ cells, expressed as frequencies of live cells and normalized to 100%. NK cell differentiation stages are presented as percentages of their respective parent populations. The middle ring shows subsets based on CD49b and CD11b expression. The outer ring further subdivides CD49b^+^CD11b^+^ NK cells according to terminal differentiation markers KLRG1 and CD11b. Bar graphs show the frequency of sequential NK differentiation stages, from pre- CD122^+^CD3^−^NKp46^−^ precursor-like compartment to highly mature NK cells (as defined in Table 5). All the data presented in the bar graphs represent the arithmetic mean ± standard error of the mean (n = 3). Red arrows indicate lineage progression from parent populations. nHT, naïve helper T helper cells; cmHT, central memory helper T cells; efmHT, effector memory helper T cells; efHT, effector helper T cells; nCT, naïve cytotoxic T cells; cmCT, central memory cytotoxic T cells; efmCT, effector memory cytotoxic T cells; efCT, effector cytotoxic T cells.

- Perform instrument calibration according to the manufacturer’s instructions. Generate the compensation matrix using unstained and single-stain control samples and calculate the compensation values.
- Establish the gating strategy (Figure 3) to identify the cell subsets of interest, ensuring proper subset hierarchy and marker exclusivity.
- Start by excluding debris based on forward scatter (FSC) and side scatter (SSC) parameters, followed by doublet discrimination and dead cell exclusion.
- Gate the specific immune populations by excluding unwanted cell populations and selecting the desired subsets (Table 5; Figure 4) (see Note 12).
- Acquire and record samples (see Note 13).
- For immunophenotyping, acquire samples with sufficient events to allow reliable analysis of rare populations (e.g., T cell subsets or NK cell maturation stages), ensuring that the rarest population is represented by at least 100 events [42]. Representative event counts obtained using the present protocol, together with cell yield and viability data, are provided in Supplementary Table S1.
- (Optional) If required, acquire additional events from the same sample to improve representation of rare or previously uncharacterized populations. The newly acquired data can be appended to the original dataset to increase total event counts.
- Export .fcs files for downstream analysis in FlowJo or equivalent FC analysis software.
- (Optional) For quantitative analysis, export population frequencies as percentages of either total live cells or the respective parent population, depending on the desired data representation (see Figure 5 for an example).

### 3.6. Notes

1. The 1× RBC lysis step is optional and should be performed only when a high number of erythrocytes is present (e.g., when the excised lymph node appears visibly bloody). Note that this step may also result in the loss of immune cells.
2. Fixation Buffer is used to preserve cellular morphology and stabilize fluorescence signals by crosslinking proteins, allowing delayed acquisition and improved sample handling. However, fixation is not required if samples are acquired promptly after staining. In most cases, stained cells can be reliably measured within 1–2 h when kept on ice and protected from light. Prolonged delays without fixation may result in decreased signal intensity and reduced cell viability.
3. For flow cytometer configurations other than that shown in Table 2, appropriate compensation must be applied to correct for fluorochrome spillover. Changes in instrument configuration may also require verification and optimization of antibody titrations.
4. Clean the glass cutting board with distilled water, followed by 70% ethanol using sterile gauze. Allow the glass cutting board to air-dry for 1 min.
5. Mechanical homogenization increases the surface area of the tissue, thereby accelerating enzymatic digestion of connective tissue by increasing collagenase and hyaluronidase activity. The same scalpels may be reused between lymph node samples, provided they are sterilized with 70% ethanol and air-dried between uses.
6. To reduce the overall procedure time, preheat the thermoshaker to 37 °C before starting. Place the 50 mL centrifuge tube in the thermoshaker at a 60-degree angle to ensure optimal enzyme distribution.
7. Wash the cell strainer sequentially. First, rinse the strainer with 1 mL of 1× PBS, then gently press the tissue against the strainer membrane using a syringe plunger. Repeat this process four times to maximize cell recovery.
8. The expected number of cells from the 11 isolated lymph nodes should amount to approximately 50 × 10^6^ cells with high viability.
9. All FACS tubes should contain the same number of cells. If insufficient cells are available, reduce the total number of cells per tube accordingly. To obtain reliable results, a minimum of 1 × 10^6^ cells per tube is required.
10. Pool cells from each sample and distribute the same number of cells used for the sample tubes into the unstained control, the live/dead single-stain control, and each FMO control tube.
11. Fc receptor blocking (e.g., TruStain FcX™ Plus) is recommended to reduce non-specific antibody binding to Fc receptors (e.g., CD16/CD32) on immune cells, thereby improving staining specificity and signal resolution. This is particularly important when working with myeloid and NK cell populations.
12. The Fixable Viability Dye eFluor^®^ 780 is membrane-impermeant and stains only cells with compromised membranes; therefore, the negative population should be gated as the live cell subset.
13. Vortex samples briefly immediately before acquisition to ensure a homogeneous cell suspension and prevent cell settling, which may affect data consistency.

## 4. Expected Results and Discussion

This protocol is optimized for identification of maturation stages of T lymphocytes and NK cells in mouse lymph nodes from BALB/cAnNCrl mice. The key to successful isolation and downstream immune cell characterization depends on the speed and quality of lymph node excision, which directly influence cell viability and preservation of surface epitopes. The protocol was optimized for the specified eleven lymph nodes (presented in Figure 2 and Table 3) due to their anatomical location and relatively easy identification. Application of the protocol to three independent biological replicates yielded 52.1 ± 11.0 × 10^6^ cells per mouse with a viability of 98.4 ± 1.3%. Representative protocol performance metrics, including cell yield, viability, and event counts for all the immune cell populations defined in Table 5, are summarized in Supplementary Table S1. From the aspect of the research goal and if the required total number of immune cells is lower, the type and number of lymph nodes can be reduced, which can also help to speed up the isolation and thereby ensure higher viability of the extracted immune cells. However, it should be noted that the same set of lymph nodes should always be collected to preserve consistency between treatment and control samples.

In some experimental settings, LPS may be administered as a systemic inflammatory stimulus to induce robust innate immune activation and to assess its impact on lymphocyte maturation and activation states. LPS triggers activation of innate immune pathways, leading to cytokine release (e.g., IL-12, IL-18, and type I interferons) and subsequent activation and redistribution of immune cells, including NK cells and T lymphocytes. Following LPS administration, mice may exhibit transient clinical signs such as reduced activity, piloerection, weight loss, and mild hypothermia. While mortality is generally limited (approximately 15% under typical experimental conditions), appropriate monitoring and supportive care are essential. To promote recovery, mice should be closely observed and provided with supportive measures such as accessible food and water, minimized handling stress, and external heat support to maintain body temperature.

Another crucial step is the handling of the samples during lymph nodes dissociation and single cell suspension preparation. Throughout the whole process the cells should be handled gently and kept on ice to prevent phenotypic changes in the isolated cells. Slow and harsh workflow and/or inappropriate cell processing can rapidly decrease the viability of cells and may also negatively affect the FC data, as antigen/epitope may degrade. In case of a higher number of mice per group, high-throughput tissue isolators should be considered, with potential cell loss and cross-sample contamination carefully assessed beforehand.

At the cell-counting step, a small aliquot of the cell suspension may be taken for a rapid viability assessment using propidium iodide (PI). PI selectively labels cells with compromised membranes, thereby identifying dead or damaged cells, which can be quantified by examining several fields of view under a fluorescence microscope. Because murine lymph nodes typically yield highly viable single-cell suspensions, total cell counting is generally sufficient for determining the number of cells required for downstream staining. Viability assessment is therefore optional and serves primarily as a quality-control measure to verify successful tissue dissociation and sample handling. Cell viability is subsequently assessed during flow cytometric analysis using a fixable viability dye, allowing exclusion of dead cells from all downstream analyses. If performed, PI-based viability assessment should be carried out in parallel with the general protocol to minimize sample processing time and preserve cell viability. Because multiparametric flow cytometry-based phenotyping often involves fluorophores with overlapping emission spectra, generation of an accurate compensation matrix is essential. To determine baseline autofluorescence and enable correct quantification, an unstained control sample is also required. These challenges can be mitigated by using spectral FC, which measures the full emission spectrum of each fluorochrome across all lasers [43], or CyTOF-based approaches, which employ isotope-conjugated antibodies and therefore do not require fluorescence compensation [44]. Nevertheless, even with these platforms, appropriate controls such as single-stain controls remain necessary. For reliable immunophenotyping and quantitative analysis, well-defined gating strategies and proper assessment of non-specific antibody binding are critical, which is facilitated by FMO controls.

A notable strength of the presented protocol is the integration of T cell differentiation and NK cell maturation analysis within a single multicolor flow cytometry panel. While numerous studies have described panels focused either on T cell memory and activation states or on NK cell developmental and maturation stages, these populations are typically analyzed separately and often require independent staining panels. By combining markers that resolve CD4^+^ and CD8^+^ T cell differentiation together with sequential NK cell maturation stages, the present protocol enables simultaneous characterization of adaptive and innate lymphocyte compartments from the same lymph node sample. To our knowledge, this specific marker combination has not been previously described.

The gating strategy for the presented multicolor FC panel (Figure 3 and Figure 4; Table 5) is based on sequential exclusion of debris, doublets, dead cells, and unwanted lineages, followed by identification of targeted lymphocyte subsets. In this protocol, particular emphasis is placed on characterization of CD4^+^ and CD8^+^ T cell subsets and NK cell maturation stages after two intraperitoneal LPS injections (day 0 and day 13), as outlined in Figure 1. The dataset presented in Figure 5 is provided as an example of panel application and data visualization, illustrating how the identified immune populations and their differentiation states can be represented using the proposed gating strategy. The presented data are intended to demonstrate resolution of the analyzed populations and should not be interpreted as a formal assessment of LPS-induced immune remodeling.

Specifically, the panel allows stratification of CD4^+^ and CD8^+^ T cells into naïve, effector, and memory states based on CD62L and CD44 expression, which can be further characterized using the terminal differentiation-associated markers CD11b and KLRG1. Nevertheless, these classifications should be interpreted within the biological context of the experiment, as the marker combinations employed define phenotypic rather than strictly functional states. For example, the CD62L^−^CD44^−^ population designated as “effector” may not exclusively represent antigen-experienced effector T cells, while CD11b and KLRG1 expression can be influenced by multiple developmental and activation-related processes.

NK cells were first identified within the CD45^+^CD3^−^ leukocyte compartment based on expression of NKp46, which defines NK lineage cells. Prior to NKp46 acquisition, a precursor-like compartment (Stage 1) was operationally defined as CD3^−^CD122^+^CD244^−^/^+^ cells lacking NKp46 expression and was included to capture cells with features consistent with early NK lineage specification. It should be noted that this population may contain heterogeneous lymphoid precursors because the panel does not include lineage exclusion or progenitor-specific markers such as CD127, CD117, or CD135. Consequently, Stage 1 should not be interpreted as a purified NK progenitor population or as directly corresponding to established NK developmental stages. Acquisition of NKp46 and CD244 expression defines immature NK cells (Stage 2), corresponding to lineage-committed NK cells that had not yet acquired markers associated with peripheral maturation. Progression toward maturation was marked by the appearance of CD49b, identifying maturing NK cells (Stage 3). At this stage, the cells remained negative for late maturation markers, indicating transitional differentiation. Further maturation was characterized by acquisition of Ly49C, defining mature NK cells (Stage 4) with a fully established peripheral NK phenotype. Finally, increased expression of CD11b and/or KLRG1 identified highly mature NK cells (Stage 5), corresponding to the terminally differentiated NK cell subsets.

Importantly, as NK cells progress through maturation, the relative abundance of each successive stage typically decreases, making highly mature NK subsets inherently rare. In this panel, six differentiation-associated markers shared across T and NK cell compartments were selected for practical reasons, as they allow discrimination of major maturation stages while maintaining panel feasibility. Although this design enables robust resolution of principal subsets, inclusion of additional markers—such as those associated with terminal differentiation, activation status, or inhibitory and activating receptor repertoires—could further refine identification of intermediate and terminal NK populations. Because the most mature NK subsets represent only a small fraction of total NK cells, deeper characterization of their functional capacity and potential memory-like properties may benefit from integration of intracellular, transcriptional, or secreted markers together with functional assays (e.g., cytokine production or cytotoxicity measurements).

In parallel, the panel supports detailed characterization of CD4^+^ and CD8^+^ T cell subsets and enables quantification of naïve, effector, and memory compartments across different experimental conditions. Such analyses may be valuable for studies investigating immune adaptation, trained immunity, tolerance, or other processes associated with immune modulation. Furthermore, it should be noted that NK and T cell maturation is inherently time-dependent, and expression of surface markers used to define differentiation states change dynamically, particularly under inflammatory conditions or following LPS treatment. Such temporal modulation can lead to apparent shifts in population distribution that reflect phenotypic plasticity rather than true changes in cell abundance. Interpretation of subset dynamics should therefore consider the kinetic nature of marker expression.

Although this 14-marker panel is optimized for NK and T cell subset analysis, it can be modified to identify additional immune populations, such as neutrophils or monocytes, if required for specific research questions. However, such modifications would require careful optimization of fluorochrome combinations and marker selection to preserve resolution of rare lymphocyte subsets. The panel can also be adapted for use in other mouse strains—for example, by substituting NKp46 with NK1.1 when analyzing strains such as C57BL/6.

For quantitative analysis, population frequencies may be expressed as percentages of total live cells to monitor global immune shifts, or relative to parent populations to assess marker expression within defined subsets. Overall, this protocol provides a robust framework for high-resolution immunophenotyping of T and NK cell compartments. By integrating analysis of T cell differentiation and NK cell maturation into a single assay, it enables comprehensive characterization of adaptive and innate lymphocyte responses while preserving limited lymph node material. The protocol can be applied to diverse experimental settings, including inflammation models, infection studies, cancer immunology, and therapeutic intervention studies.

## Figures and Tables

**Table 1:** Fluorescently labeled antibodies and viability dye for immunophenotyping.

Cell Staining Antibody	Conjugate	Clone	Dilution	Stock Conc. (µg/µL)	Manufacturer	Cat. N.
rat anti-mouse CD11b	BUV395	M1/70	1:64	0.2	BD Biosciences	563553
rat anti-mouse CD44	BUV496	IM7	1:64	0.2	BD Biosciences	741057
rat anti-mouse CD3	BUV615	17A2	1:32	0.2	BD Biosciences	751418
hamster anti-mouse KLRG1	BUV737	2F1	1:64	0.2	BD Biosciences	741812
rat anti-mouse CD122	Super Bright™ 436	TM-b1	1:32	0.2	eBioscience	62-1222-82
rat anti-mouse CD4	BV480	RM4-5	1:128	0.2	BD Biosciences	565634
rat anti-mouse CD244	mFluor Violet 610 SE	244F4	1:120	0.75	Novus Biologicals, Centennial, CO, USA	NBP2-00223MFV610
rat anti-mouse CD8	NovaFluor™ Blue 510	53-6.7	1:32	0.1	eBioscience	M003T02B01-A
rat anti-mouse CD62L	NovaFluor™ Blue 610-30S	MEL-14	1:160	0.1	eBioscience	M006T02B05-A
rat anti-mouse CD49b	PerCP-eFluor™ 710	DX5	1:64	0.5	eBioscience	46-5971-82
syrian hamster anti-mouse Ly-49C	PE	14B11	1:64	0.2	Biolegend	108208
rat anti-mouse CD45	NovaFluor™ Yellow 610	30-F11	1:160	0.1	eBioscience	M005T02Y03-A
rat anti-mouse NKp46	PE/Cyanine7	29A1.4	1:64	0.2	Biolegend	137618
Fixable Viability Dye	eFluor^®^ 780	n/a	1:1000	/	eBioscience	65-0865-14

**Table 2:** Configuration of the BD FACSymphony A3 flow cytometer used for the described flow cytometry panel.

Laser	Filter	Marker
UV	UV-379/28	CD11b
	UV-515/30	CD44
	UV-580/20	
	UV-610/20	CD3
	UV-670/25	
	UV-735/30	KLRG1
	UV-810/40	
Violet	V-431/28	CD122
	V-525/50	CD4
	V-586/15	
	V-605/40	CD244
	V-677/20	
	V-710/50	
	V-750/30	
	V-810/40	
Blue	B-530/30	CD8
	B-610/20	CD62L
	B-670/30	
	B-710/50	CD49b
	B-750/30	
	B-810/40	
Yellow/ Green	YG-586/15	Ly-49C
	YG-610/20	CD45
	YG-670/30	
	YG-780/60	NKp46
Red	R-670/30	
	R-730/45	
	R-780/60	Live/Dead

**Table 3:** Overview of lymph node sites collected per mouse, including laterality and total count. Bilateral lymph nodes were collected from both left and right sides, while mesenteric lymph nodes were counted as single units.

Lymph Node Type	Laterality	N per Mouse
Accessory axillary	Bilateral	2
Proper axillary	Bilateral	2
Subiliac (inguinal)	Bilateral	2
Lateral iliac	Bilateral	2
Colic	Single	1
Jejunal	Single	1
Caudal mesenteric	Single	1
**Total**		11

**Table 4:** Overview of the sample and control tubes required for FC. The number of immunophenotyping samples depends on the experimental design. FMO controls should be prepared for each marker in the staining panel, and single-stain compensation controls should be included for each fluorochrome-conjugated antibody and the live/dead dye.

Category	Tube/Control Type	Number Required	Purpose/Comment
**Experimental samples**	Immunophenotyping samples	Variable	One tube per biological sample and staining panel. Total number depends on experimental conditions.
**Negative control**	Unstained control	1	Used to assess autofluorescence and background signal.
**FMO controls**	One FMO control per marker in the panel	13	Each FMO contains all reagents except one antibody and is used to define gating boundaries for dim or overlapping populations.
**Viability control**	Positive live/dead single-stain control	1	Prepared using dead cells and stained only with the live/dead dye. Used to set the viability gate.
**Compensation controls**	Single-stain compensation control for each antibody fluorochrome	13	Prepared using compensation beads stained with individual antibody per tube. Used to calculate the compensation matrix.
**Compensation controls**	Single-stain compensation control for live/dead dye	1	Used to include the viability dye in the compensation matrix.

**Table 5:** Phenotypic cell markers of particular immune populations.

Cell Population	Cell Surface Markers
**Live cells**	Fixable Viability Dye eFluor 780^−^
**Immune cells**	CD45^+^
**Lymphocytes**	CD45^+^ CD3^+^
**CD4^+^ T cells**	CD45^+^ CD3^+^ CD4^+^
**Naive helper T cells (nHT)**	CD45^+^ CD3^+^ CD4^+^ CD62L^+^ CD44^−^
**Central memory helper T cells (cmHT)**	CD45^+^ CD3^+^ CD4^+^ CD62L^+^ CD44^+^
**Effector memory helper T cells (efmHT)**	CD45^+^ CD3^+^ CD4^+^ CD62L^−^ CD44^+^
**Effector helper T cells (efHT)**	CD45^+^ CD3^+^ CD4^+^ CD62L^−^ CD44^−^
**CD8^+^ T cells**	CD45^+^ CD3^+^ CD8^+^
**Naive cytotoxic T cells (nCT)**	CD45^+^ CD3^+^ CD8^+^ CD62L^+^ CD44^−^
**Central memory cytotoxic T cells (cmCT)**	CD45^+^ CD3^+^ CD8^+^ CD62L^+^ CD44^+^
**Effector memory cytotoxic T cells (efmCT)**	CD45^+^ CD3^+^ CD8^+^ CD62L^−^ CD44^+^
**Effector cytotoxic T cells (efCT)**	CD45^+^ CD3^+^ CD8^+^ CD62L^−^ CD44^−^
**NK cells**	CD45^+^ CD3^−^ CD122^+^ NKp46^+^
**CD3^−^CD122^+^CD244^−^/^+^NKp46^−^ precursor-like compartment (Stage 1** **)**	CD45^+^ CD3^−^ CD122^+^ CD244^−/+^ NKp46^−^
**Immature NK cells (Stage 2)**	CD45^+^ CD3^−^ CD122^+^ CD244^−/+^ NKp46^+^ CD49b^−^ Ly-49C^−^
**Maturing NK cells (Stage 3)**	CD45^+^ CD3^−^ CD122^+^ CD244^+^ NKp46^+^ CD49b^+^ Ly-49C^−^
**Mature NK cells (Stage 4)**	CD45^+^ CD3^−^ CD122^+^ CD244^+^ NKp46^+^ CD49b^+^ Ly-49C^+^ CD11b^−^ KLRG1^−^
**Highly mature NK cells (Stage 5)**	CD45^+^ CD3^−^ CD122^+^ CD244^+^ NKp46^+^ CD49b^+^ Ly49C^+^ CD11b^+^ and/or KLRG1^+^

## Data Availability

All the data generated or analyzed during this study are included in this manuscript.

## Supplementary Materials

The following supporting information can be downloaded at https://www.mdpi.com/article/10.3390/mps9030097/s1. Supplementary Table S1: Cell yield, viability, and event counts for immune cell populations identified using the presented flow cytometry panel (n = 3 biological replicates).
