# Supplementary material for: Multiparametric Flow Cytometry Panel for Characterization of Mouse T Cell Differentiation and NK Cell Maturation Following Inflammatory Challenge
Source: Methods Protoc. 2026 Jun 12;9(3):97. doi: 10.3390/mps9030097 (PMC13305854; doi:10.3390/mps9030097)
Supplement: Supplementary file 1 [file mps-09-00097-s001.zip › mps-4327838-supplementary.pdf]

Supplementary Table S1. Cell yield, viability, and event counts for immune cell populations identified using the presented flow cytometry panel (n = 3 biological replicates).

**Mouse ID**	**Yield (N of isolated cells)**	**Viability (% live cells within singlets)**	**nHT**	**cmHT**	**efmHT**	**efHT**	**nCT**	**cmCT**	**efmCT**	**efCT**
M00	55400000	99,2	2729	1228	4222	10164	2601	663	1534	7577
M01	61100000	99	154148	6837	15253	30958	47800	3306	1687	15618
M10	39800000	96,9	15588	781	2516	22203	3920	284	646	7842
**Mean**	52100000	98,37	57488	2949	7330	21108	18107	1418	1289	10346
**St. dev.**	11026786	1,27	83956	3375	6914	10440	25723	1646	562	4568

**Mouse ID**	**Precursor Stage 1**	**NK Stage 2**	**NK Stage 3**	**NK Stage 4**	**NK Stage 5 CD11b+KLRG1-**	**NK Stage 5 CD11b-KLRG1+**	**NK Stage 5 CD11b+KLRG1+**
M00	168	135	548	200	140	131	352
M01	1523	121	3187	932	782	189	1646
M10	217	176	895	358	135	136	406
**Mean**	636	144	1543	497	352	152	801
**St. dev.**	769	29	1434	385	372	32	732

Population abbreviations (nHT, cmHT, efmHT, efHT, nCT, cmCT, efmCT, efCT, Precursor Stage1, NK Stage 2, NK Stage 3, NK Stage 5 CD11b+KLRG1-, NK Stage 5 CD11b-KLRG1+, and NK Stage 5 CD11b+KLRG1+) and their corresponding full names and phenotypic marker definitions are described in Table 5 of the main manuscript.
